# The use of specific para-clinical examinations in patients presenting with dyspnea

**DOI:** 10.1186/1757-7241-23-S1-A24

**Published:** 2015-07-16

**Authors:** Marie Krogh Nielsen, Anja Strehlau, Mikkel Brabrand

**Affiliations:** 1 Department of Medicine, Sydvestjysk Sygehus, Esbjerg, Denmark; 2 Department of Medicine, OUH Svendborg Sygehus, Svendborg, Denmark; 3 Department of Emergency Medicine, OUH Odense University Hospital, Odense, Denmark

## Background

A large, and growing, number of patients are admitted and treated acutely at emergency departments. In Denmark, the number of acute admissions has increased 15% from 2006 to 2012. The Region of Southern Denmark has begun a process to prepare a regional concept for the Emergency Departments. Therefore, it is of interest to observe our behaviour as physicians in the use of para-clinical examinations. If every patient who enters the emergency department with dyspnea ends up having a chest X-ray, arterial blood gas analysis (BGA), or electrocardiogram (ECG) performed, the natural consequence would be to do them initially at arrival. We performed the present study with the objective to quantify the use of specific examinations on patients presenting with identical primary complaints.

## Methods

We conducted this as a prospective observational cohort study. All patients admitted at the medical admission unit at the Hospital of South West Jutland from 1 July until 31 October 2012 were registered with their subjective primary complaint by the admitting nurse. From the department of radiology and the department of clinical biochemistry, data was extracted on all examinations performed, and matched with the registered individuals.

## Results

We registered 5,966 contacts (4,782 individual patients). Of these, 946 (15.8%) presented dyspnea as the primary complaint. 56.8% had chest X-rays performed, 61.1% had blood gas taken and 73.9% had performed electro-cardiography within the first 24 hours of admittance. Furthermore, standard biochemistry including haemoglobin (hgb), creatinine (crea), and C-reactive protein (CRP), was measured in 81.2% of the cases. Blood culture tests were done in 36.8% of admissions. There was no significant difference in the use of para-clinical examinations when patients where readmitted during the observation period.

## Conclusion

When planning the admission process at the medical admission unit, attention should be paid to the fact that not all patients with acute dyspnea are, according to the admitting doctors, in need of an acute X-ray, BGA, ECG, or blood samples. If all patients presenting with dyspnea should be examined including these parameters, it would require a significant extra capacity.

